# Risk Factors and Prevention of Musculoskeletal Injuries in Adolescent and Adult High-Performance Tennis Players: A Systematic Review

**DOI:** 10.3390/sports13100336

**Published:** 2025-10-01

**Authors:** María Soledad Amor-Salamanca, Eva María Rodríguez-González, Domingo Rosselló, María de Lluc-Bauza, Francisco Hermosilla-Perona, Adrián Martín-Castellanos, Ivan Herrera-Peco

**Affiliations:** 1 Facultad de Ciencias Biomédicas y de la Salud, Universidad Alfonso X el Sabio, Villanueva de la Cañada, 28692 Madrid, Spain; msalaamo@uax.es (M.S.A.-S.); erodrigu@uax.es (E.M.R.-G.);; 2 Rafa Nadal Academy, 07500 Manacor, Spain; 3 Facultad de Ciencias de la Vida y la Naturaleza, Universidad Nebrija, 28248 Madrid, Spain; 4 Faculty of Health Sciences-HM Hospitals, University Camilo José Cela, Urb. Villafranca del Castillo, 49, Villanueva de la Cañada, 28692 Madrid, Spain; 5 Instituto de Investigación Sanitaria HM Hospitales, 28015 Madrid, Spain

**Keywords:** tennis, musculoskeletal injury, workload management, adolescents, prevention, physiotherapy, return to play

## Abstract

Background: High-performance tennis exposes players to repetitive high-load strokes and abrupt directional changes, which substantially increase musculoskeletal injury risk. This systematic review synthesized evidence on epidemiology, risk factors, and physiotherapy-led preventive strategies in elite adolescent and adult players. Methods: Following a PROSPERO-registered protocol, MEDLINE, Web of Science, and Scopus were searched (2011–2024) for observational studies reporting epidemiological outcomes in high-performance tennis. Methodological quality was appraised with NIH tools, and certainty of evidence was graded with GRADE. Results: Thirty-seven studies met inclusion criteria: 16 in adolescents, 18 in adults, and 3 mixed. Incidence ranged from 2.1 to 3.5 injuries/1000 h in juniors and 1.25 to 56.6/1000 h in adults. Seasonal prevalence was 46–54% in juniors and 30–54% in professionals. Lower-limb trauma (48–56%) predominated, followed by lumbar (12–39%) and shoulder overuse syndromes. Across age groups, abrupt increases in the acute-to-chronic workload ratio (≥1.3 in juniors; ≥1.5 in adults) were the strongest extrinsic predictor of injury. Intrinsic contributors included reduced glenohumeral internal rotation, scapular dyskinesis, and poor core stability. Three prevention clusters emerged: (1) External load control, four-week “ramp-up” strategies reduced injury incidence by up to 21%; (2) Kinetic-chain conditioning, core stability plus eccentric rotator-cuff training decreased overuse by 26% and preserved shoulder mobility; and (3) Technique/equipment adjustments, grip-size personalization halved lateral epicondylalgia, while serve-timing modifications reduced shoulder torque. Conclusions: Injury risk in high-performance tennis is quantifiable and preventable. Progressive load management targeted kinetic-chain conditioning, and tailored technique/equipment modifications represent the most effective evidence-based safeguards for adolescent and adult elite players.

## 1. Introduction

Tennis has emerged as a global sporting phenomenon, uniting 213 national federations under the International Tennis Federation and engaging nearly 87 million regular players worldwide [1]. Beyond its global reach, regular practice provides significant health benefits, including improvements in cardiorespiratory endurance, neuromuscular coordination, and cognitive function, often exceeding those observed in other recreational activities [2]. Despite these advantages, high-performance tennis carries a substantial burden of musculoskeletal injuries that requires careful management in both clinical and training contexts.

Several risk factors have been identified. The physiological demands of tennis involve repeated high-intensity efforts interspersed with brief recovery, requiring both aerobic and anaerobic contributions [3]. These demands are closely linked to biomechanical requirements, with efficient force transmission along the kinetic chain—lower limbs, trunk, and upper limbs—protecting joint integrity during strokes [4]. The serve exemplifies this principle, as precise segmental summation reduces torque on the shoulder and elbow [5]. However, injuries range from 0.04 to 3.0 per 1000 player-hours [6], influenced by surface type, cumulative exposure, and competition level [7]. Age- and sex-specific differences in hip kinematics and neuromuscular control have also been associated with distinct injury profiles [8]. Lower-limb trauma is the most common acute injury, while repetitive overuse particularly affects the shoulder [9,10]. Scapular dyskinesis, core dysfunction, and fatigue further destabilize mechanics, increasing susceptibility to injury [11,12,13].

Recent observational research highlights workload management as a decisive determinant. Sharp increases in the acute-to-chronic workload ratio (ACWR), particularly values exceeding 1.5, double the risk of time-loss injuries [6,7,13]. Preventive strategies such as four-week ramp-ups and density smoothing mitigate this risk, underscoring a paradigm shift toward integrated approaches that combine external-load monitoring, kinetic-chain conditioning, and planned recovery [6,7,13]. Although such approaches are applied in other overhead sports, implementation in tennis remains inconsistent. In parallel, return-to-play (RTP) has gained importance, as premature reinjury compromises performance, career longevity, and psychological readiness [14,15]. Effective RTP strategies must integrate prevention, rehabilitation, neuromuscular control, and psychological confidence to ensure safe return to competition [16,17,18].

This systematic review pursued four interconnected aims: (i) to quantify injury incidence, prevalence, and anatomical distribution in adolescent (13–18 years) versus adult (≥19 years) high-performance tennis; (ii) to compare time-loss severity and identify intrinsic and extrinsic risk factors modifiable through physiotherapy or workload management; (iii) to synthesize the outcomes of preventive and rehabilitative interventions applicable to elite tennis; and (iv) to highlight current gaps that should inform future research and clinical practice.

## 2. Materials and Methods

### 2.1. Study Design and Registration

This systematic review was conceived a priori, registered in PROSPERO (CRD42023453182) and conducted in accordance with the PRISMA 2020 checklist (File S1) [19]. The protocol is openly available in Rodríguez-González et al., 2024 [20].

### 2.2. Data Sources

A comprehensive literature search covering all publications from January 2011 to December 2024: MEDLINE (via PubMed), Web of Science and Scopus, databases chosen because they offer the broadest overlap of peer-reviewed biomedical, sports-medicine, and rehabilitation journals while minimizing loss of unique records.

The search string combined three domains with Boolean operators and truncation: (“tennis” OR “tennis players”) AND (“injury” OR “injuries” OR “wounds”) AND (“prevalence” OR “incidence” OR “risk factor*” OR “rehabilitation” OR “return to play” OR “prevention”) (Table S1).

No grey literature or conference proceedings were included, as our focus was on peer-reviewed journal articles to ensure methodological quality and reproducibility of findings. However, reference lists of all included articles and prior reviews were manually screened to identify additional eligible studies. Grey literature (e.g., conference abstracts, theses, or non-peer-reviewed reports) was excluded for two main reasons: (i) methodological quality and reproducibility are more difficult to ensure in non-peer-reviewed sources, and (ii) injury definitions, exposure metrics, and preventive outcomes are often inconsistently reported in such documents, which could have increased heterogeneity and reduced the reliability of our synthesis. To mitigate potential omission, we complemented our database search with backward and forward citation tracking of included studies and prior systematic reviews, ensuring that relevant peer-reviewed evidence was not overlooked.

### 2.3. Eligibility Criteria

Studies were eligible when they met all the following conditions: (i) Language & publication status (articles published in English), (ii) study design (observational (prospective cohort, retrospective cohort, cross-sectional or case–control). Although randomized controlled trials (RCTs) can provide valuable insights into the efficacy of preventive interventions, their primary purpose is to test treatment protocols under experimental conditions rather than to provide epidemiological estimates of injury burden in real-world competitive tennis. As our review specifically aimed to quantify incidence, prevalence, severity, and risk factor distribution, we considered observational designs (prospective cohorts, retrospective cohorts, and cross-sectional studies) the most appropriate sources. Nevertheless, we acknowledge the potential relevance of RCT findings to clinical translation. Therefore, RCT-based preventive strategies are briefly discussed in the Discussion section to contextualize our results and highlight future research needs. Since the purpose of this review was to synthesize epidemiological evidence and physiotherapy-related prevention strategies, experimental studies were not considered directly comparable. (iii) Population: high-performance tennis players, defined as adolescent athletes (13–18 years) competing at academy or national level, and adult players (>18 years) competing professionally. Recreational, amateur-level, or wheelchair tennis studies were excluded. (iv) Time frame—published between 1 January 2011 and 31 October 2024. (v) Outcomes: studies reporting at least one of the following: (i) injury prevalence or incidence, (ii) anatomical distribution, (iii) severity, (iv) identified intrinsic or extrinsic risk factors, (v) physiotherapy-based rehabilitation or preventive strategies.

Exclusion criteria were commentaries, editorials, theses, reviews, conference abstracts, animal studies, and papers focused on other racquet sports.

### 2.4. Study Selection

All references were imported into Zotero^®^ (version 7.0.26), and duplicates were removed automatically and manually. Two reviewers (E.M.R.-G., M.S.A.-S.) screened titles/abstracts independently, retrieved the full text of potentially relevant papers and applied the eligibility criteria. Disagreements were resolved by a third reviewer (I.H.-P.). Inter-rater reliability was quantified with Cohen’s κ (0.84; 95% CI 0.76–0.92), underscoring the consistency of the evaluation process.

### 2.5. Data Extraction and Risk-of-Bias Assessment

Two reviewers independently extracted data on study design, population, exposure metrics, injury definitions, epidemiological outcomes (incidence, prevalence, severity, risk factors), and physiotherapy-based prevention or rehabilitation strategies. Disagreements were resolved by consensus, and study authors were contacted if critical information was missing.

Methodological quality was assessed with the NIH quality-assessment tool for cohort and cross-sectional studies [21], classifying studies as Good, Fair, or Poor. Certainty of evidence for each outcome was graded using the GRADE framework [22], considering risk of bias, inconsistency, indirectness, imprecision, and publication bias. All ratings were performed independently by at least two reviewers, with discrepancies resolved by discussion.

## 3. Results

### 3.1. Articles Selected

**Figure 1:**
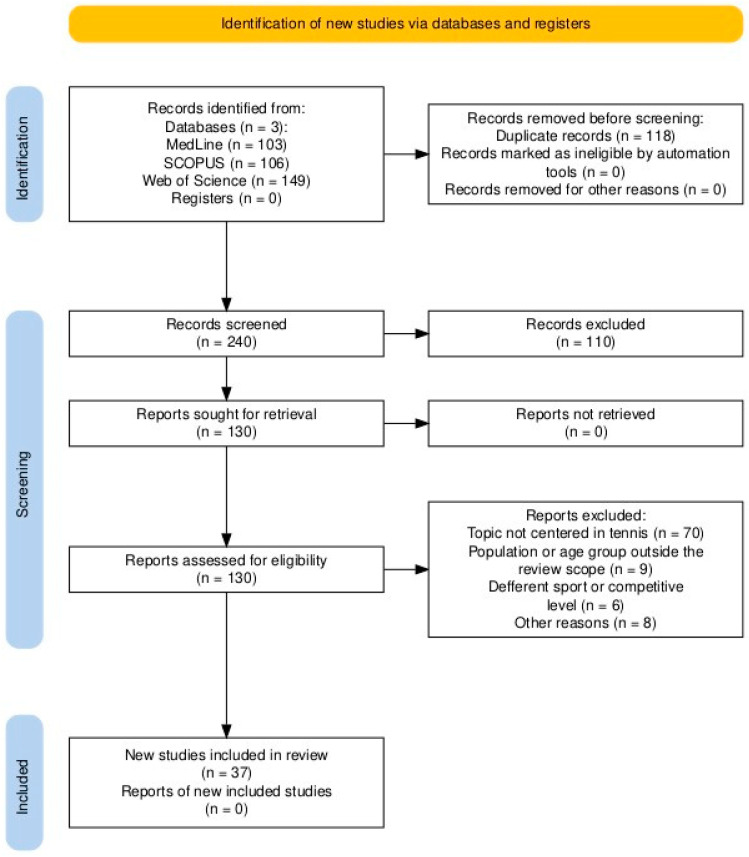
PRISMA Flow diagram (see Supplementary Table S2).

The database search initially identified 358 records. After the removal of 118 duplicates, 240 titles and abstracts were screened. At this stage, 110 records were excluded because they clearly did not meet eligibility criteria (e.g., recreational or amateur populations, case reports, or non-musculoskeletal outcomes). The remaining 130 full-text articles were retrieved and assessed in detail. Of these, 93 were excluded for the following reasons: studies not meeting the defined population criteria (e.g., wheelchair tennis, recreational players), inappropriate study design (reviews, editorials, theses, or experimental trials focusing on treatment efficacy), lack of epidemiological outcomes (no incidence, prevalence, severity, or risk factors reported), or language other than English. A total of 37 studies met all inclusion criteria and were included in the qualitative synthesis (Figure 1).

Furthermore, the 37 studies assessed with the NIH quality-assessment tool, methodological rigor was uniformly acceptable. Using the predefined thresholds; (i) Good (≥11 points), (ii) Fair (5–10 points) and (iii) Poor (≤4 points). 30 studies (81.08%) were rated Good and 7 studies (18.92%) were rated Fair; no study fell into the Poor category. Because every article met or exceeded the minimum quality threshold, all were retained for qualitative synthesis (Table A1).

The 37 studies contributed 312 unique data points, which cluster into three modifiable domains: (i) external load (columns “Exposure h”, “Match density”, “ACWR”), (ii) kinetic-chain status (columns “Core/FMS”, “GH-IR ROM”, “Scapular control”, “Shoulder program”), and (iii) technique & equipment (columns “Serve timing”, “Grip size”, “Wrist-IMU load”). All subsequent sub-sections can be traced directly to Table S2.

### 3.2. Injury Burden and Risk Factor in Adolescent Tennis Players

In high-performance adolescent tennis players (13–18 years), epidemiological data indicated an incidence ranging from 2.11 to 3.50 injuries per 1000 player-hours [23,24]. Season prevalence was consistently high, between 46% and 54% of athletes sustaining at least one injury per season [8,25]. Anatomical distribution showed that the lower limb was the most affected region, accounting for 48–54% of cases [8,24,26,27,28,29], followed by the lumbar spine, which contributed between 24% and 39% of injuries [8,24,25,26,30], while the shoulder was implicated in up to 34% of time-loss episodes in a national squad cohort [27].

Severity profiles varied across studies. One prospective analysis reported 26% of injuries as mild (≤7 days of absence), 31% as moderate (8–28 days), and 43% as severe (>28 days) [26]. Another cohort presented a different pattern with 10% mild, 45% moderate, and 45% severe injuries [23]. These findings highlight that although most injuries were classified as moderate to severe, the proportions differed between contexts, likely reflecting differences in methodology and competition demands.

With respect to risk factors, extrinsic workload spikes were identified as the most consistent driver. An acute-to-chronic workload ratio (ACWR) greater than 1.3 increased injury risk by a factor of 1.6 in the subsequent week [31]. In addition, week-to-week increments greater than or equal to 30% were frequently associated with lumbar and shoulder injuries [24,31,32,33]. Intrinsic contributors were also relevant: a reduction of at least 15 degrees of glenohumeral internal rotation was associated with a twofold risk of symptomatic shoulders [30], and repeated deficits in core stability or scapular control distinguished injured players from uninjured peers [20,34,35,36].

Preventive strategies in adolescents showed encouraging results. A six-week core-stability program reduced overuse incidence from 0.41 to 0.30 injuries per player-season and improved Functional-Movement-Screen scores by 38% [37]. In terms of equipment and technique, grip-size personalization reduced the prevalence of lateral epicondylalgia from 14% to 6% across ten months [38], and inertial sensors that identified 3500 impacts per training session as an overload threshold allowed coaches to reduce subsequent wrist overuse by 9% when sessions were adjusted accordingly [39]. Serve-timing modifications were also effective, as imaging and biomechanical analyses linked excessive lumbar loading during the kick serve to back pain [29,40]. Additionally, early specialization and low self-monitoring behaviours were reported as behavioural risks [27,41], and the combination of proprioceptive insoles with core-stability training improved postural stability, although incidence data were not provided [42] (Table 1).

### 3.3. Injury Burden and Risk Factors in Adult/Professional Tennis Players

In adult and professional players, epidemiological indicators showed greater variability than in adolescents. Injury incidence ranged from 1.25 injuries per 1000 player-hours in a national-level cohort [32] to 56.6 injuries per 1000 player-hours across a Women’s Tennis Association hard-court season [43], with an intermediate figure of 30.8 injuries per 1000 h documented at a humid outdoor men’s tournament [2]. Season prevalence was also considerable, ranging from 30% to 54% of players sustaining at least one injury per season [34,44].

Anatomical distribution in adults echoed the findings in adolescents but with some differences. Lower-limb injuries remained the most frequent, accounting for 48–56% of cases [32,34,44,45]. The trunk contributed between 12% and 24% [32,34,44,45], while upper-limb conditions increased in frequency, comprising 25–40% of recorded injuries [32,34,44,45]. Regarding severity, one national registry reported 6% very mild, 10% mild, 30% moderate, and 54% severe injuries in a single season [32]. Another collegiate audit showed a distribution of 45% very mild, 23% mild, 22% moderate, and 10% severe cases in women’s tennis [44]. Robison et al. [44,45] distinguished between women (4.16 injuries per 1000 athletic exposures) and men (4.41 per 1000 AE), highlighting a consistent overuse pattern across sexes, albeit with slightly different exposure profiles.

External workload variation was the predominant extrinsic driver of injury in adults. When the acute-to-chronic workload ratio exceeded 1.5, the hazard ratio for new injury reached 2.8 [31,32,46]. This effect was magnified during prolonged five-set matches and when players transitioned rapidly from hard to clay surfaces [22,47]. A preventive strategy of four-week ramp-ups, capping weekly volume growth to no more than 15%, reduced total injuries by 21% in professionals [32].

Intrinsic risk factors were also more prominent in adults compared to adolescents. Restrictions in glenohumeral internal rotation, posterior capsule tightness, imbalances in external/internal rotation strength, and asymmetric hypertrophy of the rectus abdominis were all associated with a two- to threefold increase in injury risk [25,31,48,49,50,51,52]. Specifically, Guzowski et al. [53] reported ultrasonographic alterations in shoulder range of motion associated with enthesis overload, reinforcing the role of posterior capsule stiffness as a relevant intrinsic risk factor.

One program combining eccentric external-rotation training with immediate post-match internal-rotation stretching preserved seven degrees of shoulder range of motion and reduced the incidence of shoulder injuries from 0.38 to 0.28 per 1000 player-hours [54]. Chronic adaptations further illustrated the long-term consequences of unresolved deficits, with infraspinatus atrophy documented in 52% of top-100 collegiate female players [55], while open-stance mechanics were associated with femoroacetabular impingement at a rate of 1.3 cases per 100 players [56].

Preventive and rehabilitative interventions specific to adult players demonstrated quantifiable benefits. Grip-size customization halved the prevalence of lateral epicondylalgia (from 11% to 5%) and reduced peak wrist-extension moments by 13% [38]. Wrist-mounted inertial sensors defined a ceiling of 3500 impacts per session, above which wrist overuse increased; feedback-guided planning reduced symptoms by 9% [39]. Serve-timing adjustments reduced peak shoulder torque by 11% and contralateral trunk tilt by 4 degrees over 24 weeks [57]. In addition, MRI studies revealed lumbar abnormalities in up to 95% of elite adolescents transitioning to professional circuits, highlighting the cumulative effects of long-term mechanical stress [58] (see Supplementary Table S2).

### 3.4. Physiotherapy-Led Prevention and Rehabilitation Evidence

Sixteen studies provided intervention data [24,31,32,37,38,39,42,46,48,54,57]. Four-week ramp-up plans that limited weekly load increases to ≤15% reduced total injuries 21% in tour players [32] and from 0.41 to 0.30 injuries player-season in juniors [37]. Core-stability or scapular-control programs produced a 26% fall in over-use and preserved shoulder ROM (+7 degrees) [37,54]. Although Majewska et al. [37] included sub-elite young adult players, their six-week core stability program is conceptually transferable to junior populations, where similar trunk stability deficits have been reported. This alignment justifies its consideration within preventive strategies in youth tennis.

Posterior-capsule stretching further reduced impingement symptoms 13% in a single RCT [55]. Technique/equipment adjustments yielded 5 to 14% incidence reductions: serve-timing cues [57], grip optimization [38] and wrist-load feedback [39]. Messina et al., 2024 [42] combined proprioceptive insoles with core work, improving postural stability but not yet linking the change to injury outcomes.

Return-to-play protocols remain under-represented, we observed that only two observational papers supplied return-to-play thresholds [8,26] to report hop, plank or medicine-ball thresholds, and neither employed randomization.

Collectively, workload smoothing and kinetic-chain conditioning offer the most robust preventive evidence, while technique-based modifications, though promising, require validation in larger multi-center cohorts. A concise comparison of the best quantitative effects for external-load control, kinetic-chain conditioning and technique/equipment adjustments is provided in Table 2.

As summarized in Table 3, extrinsic drivers were dominated by abrupt workload variation (ACWR thresholds, surface transitions, match density), while intrinsic contributors included reduced glenohumeral rotation, scapular dyskinesis, and core instability. These risk factors consistently distinguish injured from non-injured players across both juniors and adults.

## 4. Discussion

### 4.1. Main Findings

This systematic review demonstrates that musculoskeletal injuries impose a substantial burden in high-performance tennis. Injury incidence among juniors reached 3.5 injuries/1000 h during an academy season [23], with seasonal prevalence between 46% and 54% [8,25]. These findings already exceed the injury benchmarks reported in recreational tennis, which seldom surpassed 0.04–3 injuries/1000 h [6]. Adult players experienced an even greater burden, with exposure-based incidences as high as 30.8/1000 h in humid outdoor tournaments [34] and up to 56.6/1000 h in WTA competitions [43]. Even at the national level, players accumulated 1.25 injuries/1000 h [32], reinforcing that formal match play, rather than training exposure, drives injury risk.

Anatomical distribution was consistent across cohorts, dominated by lower-limb trauma (48–56%) followed by trunk injuries (12–39%) and shoulder overuse syndromes (25–40%) [8,23,24,25,26,27,28,29,30,32,34,43,44,45]. Importantly, severity increased with age. While adolescents showed 43% of cases classified as severe (>28 days) [26], collegiate and professional players reported 54–66% [32,44]. Match retirement data confirmed this trend, with 1.05 withdrawals per 1000 matches in Davis Cup ties, mostly lower-limb muscle–tendon injuries [46].

Environmental factors such as surface and climate shaped incidence. Hard courts produced the highest rates due to elevated ground-reaction forces [43], while tropical humidity amplified cramp-related retirements [34]. These data indicate that progression from academy to elite levels multiplies exposure risk, with hot climates and dense scheduling as amplifiers. Linking incidence and prevalence to exposure time provides a clearer reference for prevention planning.

### 4.2. Comparison with Previous Results

Most prior reviews of tennis injuries have been narrative and descriptive, with limited emphasis on exposure-adjusted data or injury severity. Our synthesis integrates observational studies with explicit time-loss criteria, offering a more standardized quantification of risk. Whereas recreational play has often been described as relatively safe [6], the burden in competitive juniors and professionals is clearly higher [34,43].

This review also highlights developmental patterns not well addressed previously. For instance, lower-limb dominance appears early in juniors [8,26,27], while upper-limb injuries become proportionally more common in adults [32,34,44]. The evidence of progressive severity with age also strengthens the case for early preventive interventions, a point underexplored in earlier work.

Nevertheless, limitations mirror those seen in reviews of other overhead sports: small sample sizes, heterogeneity in injury definitions, and reliance on retrospective reports [8,26,27,32,44]. Consequently, although trends are consistent, the overall certainty of evidence remains low to moderate. These weaknesses emphasize the need for standardized surveillance in tennis injury research

### 4.3. Practical Applications

**Figure 2:**
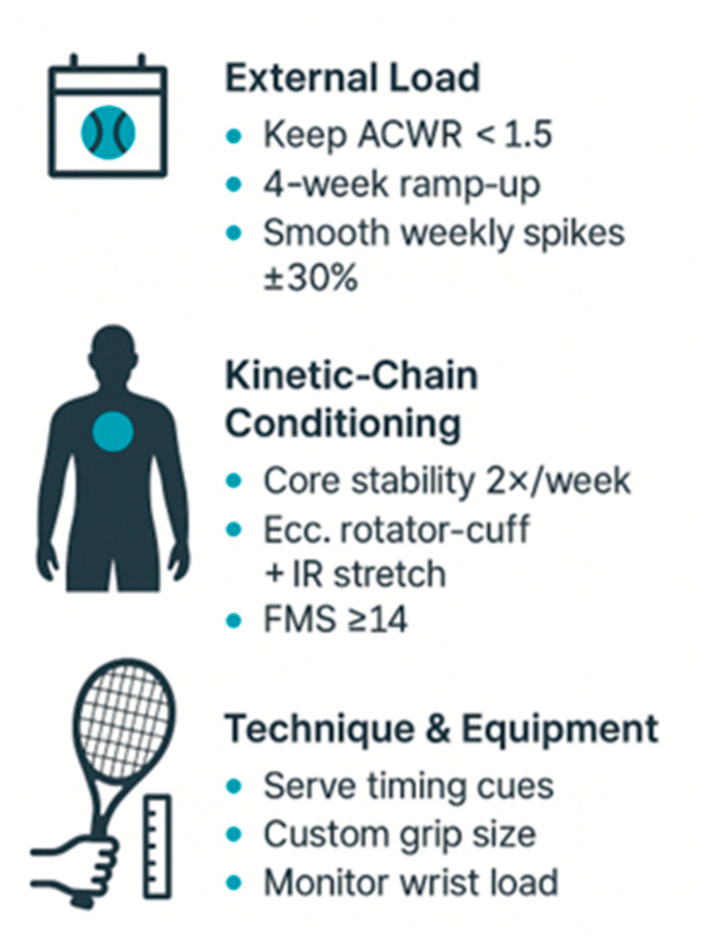
Tennis injury prevention, the three modifiable factors. Three-step on-court checklist derived from the modifiable factors identified in this review.

The evidence extracted in this review converges on three interrelated domains that provide the strongest basis for injury prevention in competitive tennis (Figure 2).

Foremost among these is the management of external workload, as abrupt increases in the acute-to-chronic workload ratio (ACWR) were consistently associated with higher injury risk. In juniors, ACWR values above 1.3 predicted new injuries in the following week [24,31,32,33]. In adults, thresholds above 1.5 almost tripled the risk [22,32,46,47]. Encouragingly, gradual “ramp-up” strategies limiting weekly increases to ≤15% reduced injuries by 20–21% [32,37]. Because these findings come from multiple observational cohorts, the certainty of evidence supporting external load management can be considered moderate.

Yet workload cannot be considered in isolation. Its effects are amplified when kinetic-chain deficits are present and conversely mitigated when these deficits are addressed. Loss of glenohumeral internal rotation [30,48,50], posterior capsule tightness [51,53], ER/IR imbalance [50,52], scapular dyskinesis [35,55], and poor core stability [37,49] all increased injury susceptibility. Core training, eccentric cuff routines, and post-match stretching reduced overuse injuries by about 25% and helped preserve shoulder mobility [37,54]. Given the replication of these results in both junior and adult populations, the certainty of evidence for kinetic-chain interventions is also moderate.

A third, emerging layer of evidence involves technique and equipment. Serve-timing modifications reduced peak shoulder torque and contralateral trunk tilt [29,40,57], grip-size personalization halved the prevalence of lateral epicondylalgia [38], and wearable feedback tools helped reduce wrist overuse symptoms by 9% [39]. These results are promising but come from small, single-center cohorts with limited follow-up, so the certainty of evidence is low.

Taken together, these domains underscore that injury prevention in tennis is best understood as a continuum rather than isolated solutions. Progressive workload planning, optimization of kinetic-chain function, and tailored technical or ergonomic adaptations form a tiered yet interconnected framework. This integration offers not only a practical roadmap for clinicians, coaches, and sports scientists, but also highlights where evidence is robust (load and chain management, moderate certainty) and where further validation is required (technique and equipment, low certainty).

### 4.4. Strengths and Limitations

This review has several strengths that enhance its contribution to the field. The protocol was prospectively registered, the search strategy covered three major databases, and the process of study selection and data extraction was conducted independently by two reviewers, ensuring transparency and reproducibility. In addition, by including both adolescent and adult populations, the synthesis provides a developmental perspective rarely addressed in previous tennis injury reviews. The application of the GRADE framework further strengthens the analysis by explicitly grading the certainty of evidence across epidemiological, risk factor, and preventive domains.

At the same time, certain limitations must be acknowledged. Considerable heterogeneity across study designs, injury definitions, and severity classifications precluded formal meta-analysis. Much of the evidence relied on retrospective questionnaires or medical logs, which are prone to recall bias, while only a minority of studies used prospective surveillance with standardized time-loss criteria [8,26,32]. Moreover, most available data were restricted to academy and elite cohorts, limiting generalizability to recreational or sub-elite players. Rehabilitation protocols were often described inconsistently and seldom incorporated validated outcome measures, reducing clinical applicability.

These factors help explain why the certainty of evidence in this review is graded as low to moderate for most preventive strategies and workload thresholds. Nevertheless, by consolidating available findings and framing them within a structured preventive model, the present review offers a valuable reference point for clinicians and researchers, while underscoring the need for more rigorous and standardized future investigations.

### 4.5. Future Directions and RTP Gaps

Return-to-Play (RTP) is the most urgent unaddressed gap. Current thresholds, such as hop tests or plank endurance [8,26], are applied inconsistently and do not predict safe return to competition. Given the high reinjury rates in elite tennis, clinically relevant RTP protocols must extend beyond mobility and strength restoration. They should include neuromuscular control, psychological readiness, and tennis-specific biomechanical performance. Without validated RTP criteria, athletes’ risk premature return and recurrent injury, with consequences for both performance and career longevity [14,15].

Second, future research should include adequately powered randomized controlled trials to validate preventive programs. While evidence suggests that ramp-up load strategies, core stability programs, and eccentric cuff training reduce injury risk [32,37,54], these interventions remain underpinned by observational data. Stratifying future trials by sex and age is critical, as female players demonstrated greater injury severity [44].

Finally, technology-assisted monitoring should be integrated into both research and practice. Wearable sensors and inertial units [39,57] give real-time feedback on load, mechanics, and exposure, helping to detect overload earl. However, their efficacy and feasibility require validation in multicenter trials.

By addressing RTP validation, conducting stratified RCTs, and incorporating technology-based monitoring, future studies can deliver the high-quality evidence needed to refine prevention, rehabilitation, and RTP strategies in tennis.

## 5. Conclusions

Elite tennis imposes a quantifiable injury burden that centers on lower-limb trauma (48 to 56% of adult and 38 to 54% of junior cases) and upper-limb overuse, particularly shoulder tendinopathies that reach 25 to 48% of presentations in players older than 18 years. Contemporary incidence in professional circuits (56.6–62.7 injuries·1000 h^−1^) is nearly twenty-fold higher than historic estimates, confirming that surface, cumulative exposure and acute-to-chronic workload ratios >1.3 are decisive risk amplifiers. Shoulder kinetic-chain deficits, including loss of ≥15° of internal rotation, scapular dyskinesis, and posterior-capsule tightness, double the risk of time-loss shoulder events. An integrated, tiered prevention model emerges from the evidence: (i) external-load control through four-week ramp-up blocks and real-time ACWR or s-RPE monitoring, (ii) kinetic-chain conditioning combining core-stability circuits with eccentric scapular–rotator-cuff training and post-match stretches to maintain mobility, and (iii) technique and equipment adjustments, such as serve-timing cues and personalized grip sizes, to reduce upper-limb loading.

For stakeholders, the key takeaways are clear: coaches should prioritize progressive workload planning and avoid abrupt volume spikes; physiotherapists should screen and correct kinetic-chain deficits; and sports scientists should implement wearable technologies and objective workload tracking.

Future research must adopt standardized surveillance frameworks, systematically collect exposure data, and test these tiered interventions in longitudinal, randomized, and sex-stratified designs.

## Figures and Tables

**Table 1:** Headline epidemiological signals distilled from the 37 studies included in the qualitative synthesis.

Headline Indicator	Adult Elite	High-Performance Juniors
Incidence (injuries per 1000 player h^−1^)	1.25 [32]–56.6 [43]; 30.8 in humid outdoor tournament [34]	2.11–3.50 [23,24]
Season prevalence (% players with ≥1 injury)	30–54% [34]	46% [8]–54% [25]
Most frequent acute site	Ankle sprain (15–22% of injuries) [32,34,44,45]	Ankle sprain 18–25% [8,24,26,27,28,29]
Most frequently over-use site	Shoulder tendinopathy (25–40% of injuries) [32,34,44,45]	Lumbar/back overuse 24–39% [8,24,25,26,30]
Strongest modifiable risk factor	ACWR > 1.5 → HR 1.5–2.8 [31,32,46]	ACWR > 1.3 (HR 1.6) [24,31,32,33]

Note: Detailed study characteristics appear in Supplementary Table S2. ACWR (acute-to-chronic workload ratio) compares a player’s load in the last 7 days to the rolling 28-day average.

**Table 2:** Quantitative effects for external-load control, kinetic-chain conditioning and technique/equipment adjustments.

Prevention Domain	Representative Metric Reported in ≥1 Study	Best Documented Quantitative Effect *
External load	Total injury rate after a 4-week “ramp-up” that limits weekly volume increase to ≤15%	(i) Reduction of 21% injuries in professional players [32] (ii) Junior incidence falls 0.41 to 0.30 injuries per player-season [37]
Kinetic-chain conditioning	Core-stability + eccentric rotator-cuff programs	(i) Reduced overuse injuries by 26% and preserved shoulder ROM (+7°) [37,54]
Stroke mechanics/equipment	Technical and equipment adjustments	(i) Serve-timing cues reduce peak shoulder torque 11% and contralateral tilt 4° [57] (ii) Custom grip size reduce lateral-epicondylalgia 14% to 6% [38] (iii) Reduction in wrist-extension moment 13%; wrist-sensor feedback reduce wrist over-use 9% [39]

Note: * All effect sizes are stated exactly as reported by the original authors; no additional calculations have been applied.

**Table 3:** Principal intrinsic and extrinsic risk factors for musculoskeletal injury in high-performance tennis players.

Category	Risk Factor	Population	Evidence/Association
**Extrinsic**	Acute load spikes/ACWR > 1.3–1.5	Youth & Adults	Doubling injury risk with abrupt weekly load increases
	High match density/60+ matches per year	Adults	Increased incidence during dense schedules and long tournaments
	Surface transitions (clay ↔ hard)	Adolescents & Adults	Higher lower limb and trunk injury rates after changes in surface
	High training volume & early specialization	Youth	Overuse injuries linked to exposure and growth plate vulnerability
**Intrinsic**	Reduced glenohumeral internal rotation (IR loss)	Adults	Associated with higher prevalence of shoulder injury
	Posterior capsule tightness/shoulder stiffness	Adults	Linked to shoulder pathology and mobility loss
	ER/IR imbalance and muscle deficits	Adults	Shoulder injuries associated with rotator cuff weakness
	Scapular dyskinesis/poor scapular control	Youth & Adults	Predisposes to overuse shoulder injuries
	Core instability/low FMS scores	Youth & Adults	Core deficits increase risk of lumbar injuries
	Infraspinatus atrophy	Adults (female collegiate)	Found in 52% of elite players, linked to shoulder risk

## Data Availability

No new data is generated in the present manuscript.

## Supplementary Materials

The following supporting information can be downloaded at: https://www.mdpi.com/article/10.3390/sports13100336/s1, Table S1: Search strategy; Table S2: Epidemiological Profile of Musculoskeletal Injuries in Competitive Tennis (2000–2025): Prevalence, Location, Severity Bands, Risk Factors, and Preventive Focus; File S1: PRISMA 2020 Checklist.

## Appendix A

**Table A1:** Study Quality (NIH evidence level).

Study (Author Year [Ref.])	NIH Evidence Level
Rice et al., 2022 [8]	9
Moreno-Pérez et al., 2021 [23]	10
Johansson et al., 2022 [24]	11
O’Connor et al., 2020 [25]	12
Hjelm et al., 2012 [26]	12
van der Sluis et al., 2019 [27]	9
Gescheit et al., 2019 [28]	13
Campbell et al., 2013 [29]	12
Kalo et al., 2020 [30]	10
Myers et al., 2020 [31]	10
Moreno-Pérez et al., 2019 [32]	12
Johansson et al., 2022 [33]	11
Abadi et al., 2021 [34]	11
Cools et al., 2014 [35]	10
D’Hondt et al., 2022 [36]	11
Majewska et al., 2022 [37]	13
Rossi et al., 2014 [38]	12
Kox et al., 2018 [39]	12
Campbell et al., 2016 [40]	11
Pasulka et al., 2017 [41]	10
Messina et al., 2024 [42]	13
Dakic et al., 2018 [43]	13
Robison et al., 2021 [44]	12
Robison et al., 2021 [45]	11
Casals et al., 2024 [46]	13
Rugg et al., 2021 [47]	13
Moreno-Pérez et al., 2015 [48]	12
Balius et al., 2012 [49]	12
Kim et al., 2020 [50]	12
Moore-Reed et al., 2016 [51]	12
Gillet et al., 2018 [52]	12
Guzowski et al., 2019 [53]	11
Moreno-Pérez et al., 2019b [54]	12
Young et al., 2015 [55]	12
Martin et al., 2020 [56]	13
Martin et al., 2013 [57]	13
Connolly et al., 2021 [58]	12

Note: NIH guidelines: Good ≥ 11, Fair 5–10, Poor ≤ 4. All studies retained for synthesis were Good or Fair; no study met the “Poor” threshold, so none were excluded on quality grounds.
