# Supplementary material for: Risk Factors and Prevention of Musculoskeletal Injuries in Adolescent and Adult High-Performance Tennis Players: A Systematic Review
Source: Sports (Basel). 2025 Oct 1;13(10):336. doi: 10.3390/sports13100336 (PMC12568103; doi:10.3390/sports13100336)
Supplement: Supplementary file 1 [file sports-13-00336-s001.zip › sports-3762294-supplementary tables.pdf]

Table S1. Search strategy

Data base	Search string
Medline	(("Injuries"[MeSH Terms] OR "Wounds and Injuries"[MeSH Terms] OR injury*[Title/Abstract] OR wound*[Title/Abstract]) AND ("Tennis"[MeSH Terms] OR "Tennis"[Title/Abstract] OR "tennis players"[Title/Abstract]) AND ("Prevention and Control"[MeSH Terms] OR prevention*[Title/Abstract] OR "Incidence"[MeSH Terms] OR incidence*[Title/Abstract] OR "Risk Factors"[MeSH Terms] OR "risk factor*"[Title/Abstract])) AND ("adolescent"[MeSH Terms] OR "adolescent"[Title/Abstract] OR "adult"[MeSH Terms] OR "adult"[Title/Abstract])
SCOPUS	TITLE-ABS-KEY (((injury* OR wound* OR "wounds and injuries") AND (tennis OR "tennis players") AND (prevention* OR incidence* OR "risk factors") AND (adolescent* OR adult*)) AND NOT (review OR "systematic review" OR "meta-analysis") AND NOT ("football" OR "basketball" OR "rugby" OR "paddle" OR "table tennis" OR "beach tennis"))
Web of Science	(injury OR injuries OR wound OR "wounds and injuries") AND (tennis OR "tennis players") AND (prevention OR incidence OR "risk factors") AND (adolescent* OR adult*) NOT ("football" OR "basketball" OR "rugby" OR "paddle" OR "table tennis" OR "beach tennis")

Table S2. Epidemiological Profile of Musculoskeletal Injuries in Competitive Tennis (2000-2025): Prevalence, Location, Severity Bands, Risk Factors, and Preventive Focus

**Study (ref.)**	**Pop. / Level**	**n / Sex**	**Injury burden**	**Main regions (%)**	**Severity (Time -loss in days)**				**Context (surface / workload)**	**Key modifiable risk factors**	**Key physio intervention**
					**0-3 (very mild)**	**4-7 (mild)**	**8-28 (moderate)**	**>28 (severe)**			
Rice et al., 2022 [8]	Adolescents–Adults	237 (53 % F)	46 % injured / yr	LL 54, TR 39, UL 11	—	—	—	—	Multi-surface; match hours	Trunk instability; low hamstring flexibility	Neuromuscular + trunk program
Moreno-Pérez et al., 2021 [23]	Youth	15 (60 % M)	3.5 / 1 000 h	LL 48, TR 25, Shoulder 18	0	10	45	45	Clay; weekly load spikes	High acute load; low chronic load	s-RPE load monitoring
Johansson et al., 2022 [24]	Youth	301 (M/F)	2.68 shoulder / 1 000 h	Shoulder 100	—	—	—	—	External load cum.	Workload peaks; spec.	Periodised load; recovery
O’Connor et al., 2020 [25]	Youth	82 (M/F)	54 % injured	Knee 19, Shoulder 17	—	—	—	—	Summer off-season spike	Acute load surge Sept.	Pre-season conditioning
Hjelm et al., 2012 [26]	Youth	55 (M/F)	100 cases / year	LL 51, UL 24, TR 24	9	17	31	43	Hard > clay; >6 h wk	Prior injury; muscle imbalance	Full rehab; return testing
van der Sluis et al., 2019 [27]	Youth	73 (M/F)	58 overuse TL	Shoulder 34, Knee 28	—	—	—	—	High exposure;	Low self-monitoring; exposure	Self-monitor education

									ACWR spikes		
Gescheit et al., 2019 [28]	Youth	101 (M/F)	Inc ↑ with age	Lumbar > Shoulder	—	—	—	—	Year-round load	Age growth; pelvis strain	Load control by age
Campbell et al., 2013 [29]	Youth	20 (M)	Lumbar pain 38 %	Lumbar 100	—	—	—	—	Kick-serve forces	Lateral flexion >8× running	Serve mechanics coaching
Kalo et al., 2020 [30]	Youth	27 (67 % M)	Shoulder deficits only	Shoulder 100	—	—	TL*	—	Indoor; years tennis	Years play; IR loss	Posterior capsule stretch
Myers et al., 2020 [31]	Youth	42 (M/F)	ACWR > 1 → HR 2.8	—	—	—	—	ACWR weekly peaks	Unprepared acute load	Four-week ramp-up	—
Moreno-Pérez et al., 2019 [32]	Adolescents–Adults	162 (M/F)	1.25 / 1 000 h	LL 38 M, TR 41 F	6	10	30	54	Clay > hard; dense schedule	Match load; surface change	—
Johansson et al., 2022 [33]	Youth.	198 (M/F)	Back pain 2.11 / 1 000 h	Lumbar 100	—	—	—	—	17 h/wk training	External load peaks	Weekly load smoothing
Abadi et al., 2021 [34]	Adults	161 (56 % M)	30.8 / 1 000 h	TR 39, LL 33, UL 25	—	—	—	61	Outdoor hard; WBGT > 28 °C	Prior injury; heat; tall; high ITF	Core/back strength; hydration

Cools et al., 2014 [35]	Youth.	59 (M/F)	No injury data	—	—	—	—	—	Scapular dyskinesia risk	MT/LT/SA strengthening	
D'Hondt et al., 2022 [36]	Youth	26 (M/F)	No injury data	—	—	—	—	—	Motor control profiles	—	
Majewska et al., 2022 [37]	Adults	160 (M/F)	—	—	—	—	—	—	Low FMS core score	Six-week core stability	
Rossi et al., 2014 [38]	Adults	8 (M)	Lateral elbow 40-50 %	Elbow 100	—	—	—	—	Grip size / impact	Excess grip force	Optimal grip size advice
Kox et al., 2018 [39]	Adults	26 (M/F)	Wrist pain 32-73 %	Wrist 100	—	—	—	—	Technique; intensity	Prior wrist pain; high load	Wrist load monitoring
Campbell et al., 2016 [40]	Youth	19 (M)	Posture risk only	—	—	—	—	—	Extreme prep posture	Core control drills	
Pasulka et al., 2017 [41]	Youth	1 190 (M/F)	44 % overuse in tennis	Tennis 58	—	—	—	—	High training volume	Early specialization; growth plates	Neuromuscular training
Messina et al., 2024 [42]	Youth	61 (M)	Postural stability link	—	—	—	—	—	Core weakness; balance ↓	Proprio insoles + core	
Dakic et al., 2018 [43]	Adults)	52 (F)	56.6 / 1 000 h	LL 51, UL 25, TR 15	57	4	—	4	Tour hard courts; 60+ matches	High rank; total matches	—

Robison et al., 2021 [44] F	Adults	– (F)	4.16 / 1 000 AE	TR 15, UL 29, LL 48	45	23	22	10	Practice > match overuse	Overuse serve mechanics	Dynamic shoulder–trunk study
Robison et al., 2021 [45]	Adults	- / (M)	4.41 / 1 000 AE	TR 15, UL 29, LL 48	—	—	—	—	Div I > III exposure	Overuse; non-contact	—
Casals et al., 2024 [46]	Adults	DavisCup 6 060 matches	Withdrawal 1.05 / 1 000	LL muscle-tendon	—	—	—	—	Hard court; 5-set ties	Long matches, fatigue	Scheduling policy guidance
Rugg et al., 2021 [47]	Adults	1 550 (M/F)	Historical trends	—	—	—	—	Era-wise exposure	Tech changes; surfaces	—	
Moreno-Pérez et al., 2015 [48]	Adults	47 (M)	Shoulder 25-48 %	Shoulder 100	—	—	—	—	Hard > clay tour	Limited IR ROM	IR stretch + ecc ER
Balius et al., 2012 [49]	Adults	61 (M/F)	RA injury 29 %	RA infra-umbilical	—	—	—	—	Serve asymmetry	RA hypertrophy non-dom.	Core eccentric control
Kim et al., 2020 [50]	Adults	58 (M/F)	Shoulder 24 %	Shoulder 100	—	—	—	—	Overhead strength	ER/IR imbalance; posterior stiffness	UQYBT screening; scap rehab
Moore-Reed et al., 2016 [51]	Adults	79 (F)	GIR ↓ in 50 %	Shoulder ROM	—	—	—	—	24 h post-match ROM	GIR loss; capsular stiff	Early IR stretch + ecc
Gillet et al., 2018 [52]	Adults	91 (M)	Shoulder injury 30 %	Shoulder ROM & strength	—	—	—	—	RE↑ IR↓; trap SA strength ↑	Muscle imbalance	Scapular strengthening

Guzowski et al., 2019 [53]	Adults	66 (M/F)	Shoulder pathology 30 %	Shoulder enthesis	—	—	—	—	ER↑, IR↓ ROM	Overload enthesis	Rotator cuff balance
Moreno-Pérez et al., 2019b [54]	Adults	26 (M)	Shoulder 8.2 / 1 000 h	Shoulder ROM changes	—	—	—	—	Single match effects	IR drop, ER strength ↓	Post-match IR stretch
Young et al., 2015 [55]	Adults	125 (F)	Infra-spinosus atrophy 52 %	Shoulder 100	—	—	—	—	NCAA hard courts	High rank workload	Scapular control screening
Martin et al., 2020 [56]	Adults	8 (M)	Hip FAI 1.3 / 100	Hip 100	—	—	—	—	Open-stance forehand	Extreme ER hip load	Hip stabilization drills
Martin et al., 2013 [57]	Adults	20 (M)	Overuse shoulder/elbow	Shoulder 100	—	—	—	—	Poor serve timing	Hyper-angulation; kinetics	Temporal serve retraining
Connolly et al., 2021 [58]	Youth	24 (M/F)	95 % lumbar MRI abn.	Lumbar	—	—	—	—	Serve kinematics	Early knee flex; pelvis rot	Serve technique cueing

Note: "—" = data not reported. Abbreviations: LL = lower limb, UL = upper limb, TR = trunk, ACWR = acute-to-chronic workload ratio, TL = time-loss.
